# Two-Dimensional Population Receptive Field Mapping of Human Primary Somatosensory Cortex

**DOI:** 10.1007/s10548-023-01000-8

**Published:** 2023-08-27

**Authors:** Michael Asghar, Rosa Sanchez-Panchuelo, Denis Schluppeck, Susan Francis

**Affiliations:** 1 Sir Peter Mansfield Imaging Centre, School of Physics and Astronomy, University of Nottingham, Nottingham, UK; 2 University Hospitals Birmingham NHS Foundation Trust, Nottingham, UK; 3 School of Psychology, University of Nottingham, Nottingham, UK; 4 NIHR Nottingham Biomedical Research Centre, Nottingham University Hospitals NHS Trust and the University of Nottingham, Nottingham, UK

**Keywords:** Population receptive fields, 7T fMRI, Primary somatosensory cortex, Primary motor cortex, Somatotopy

## Abstract

Functional magnetic resonance imaging can provide detailed maps of how sensory space is mapped in the human brain. Here, we use a novel 16 stimulator setup (a 4 × 4 grid) to measure two-dimensional sensory maps of between and within-digit (D2–D4) space using high spatial-resolution (1.25 mm isotropic) imaging at 7 Tesla together with population receptive field (pRF) mapping in 10 participants. Using a 2D Gaussian pRF model, we capture maps of the coverage of digits D2–D5 across Brodmann areas and estimate pRF size and shape. In addition, we compare results to previous studies that used fewer stimulators by constraining pRF models to a 1D Gaussian Between Digit or 1D Gaussian Within Digit model. We show that pRFs across somatosensory areas tend to have a strong preference to cover the within-digit axis. We show an increase in pRF size moving from D2–D5. We quantify pRF shapes in Brodmann area (BA) 3b, 3a, 1, 2 and show differences in pRF size in Brodmann areas 3a-2, with larger estimates for BA2. Generally, the 2D Gaussian pRF model better represents pRF coverage maps generated by our data, which itself is produced from a 2D stimulation grid.

**Supplementary Information:**

The online version contains supplementary material available at 10.1007/s10548-023-01000-8.

## Introduction

To advance the investigation of function and structure in human somatosensory areas, detailed and reproducible topographic maps of the hand representations are invaluable. Such maps can be used, for example, to study changes in amputees or patients with disorders such as focal hand dystonia (FHD), but they also enable the study of changes in the healthy brain with training and aging. Several recent 3 and 7 Tesla fMRI studies have demonstrated that human primary somatosensory cortex (S1) in individual participants can be robustly parcellated by finger (digit) representations (Besle et al. 2013; Kolasinski et al. 2016; Martuzzi et al. 2014; O’Neill et al. 2020; Sanchez-Panchuelo et al. 2012; Schweisfurth et al. 2015) (n = 6, 13, 10, 22, 6, 12 participants in each study, respectively). The ability to measure digit representations reliably in each participant is a key requisite for their use in individualised precision approaches to medicine.

Many previous studies have used so-called ‘phase-encoding’ or ‘travelling wave (TW)’ paradigms to establish the “digit dominance” of each voxel from which to generate a somatotopic map on the cortical surface (Sanchez-Panchuelo et al. 2010; Sánchez-Panchuelo et al. 2014; Schluppeck et al. 2016). Some recent studies have applied the phase-encoding design to investigate changes in the spatial representation of the cortical digit map. In complex regional pain syndrome (CRPS) patients,Mancini et al. (2019) found no difference between the affected hand and both the unaffected hand of subjects, and when compared to a control group, suggesting preservation of digit maps in CRPS patients (n = 20), whilst (Pfannmöller et al. 2019) showed a decreased representation (size) of the hand area in Brodmann Area 3b (BA3b) in the somatosensory cortex of CRPS patients. Schweisfurth et al. (2018) investigated differences between dominant and non-dominant hands, finding no differences between hemispheres (n = 12). Härtner et al. (2021) measured distances between the digit representations of D1–D5 in response to pneumatic stimulation in a block design (n = 20). They found a negative correlation between tactile acuity, as measured by a grating orientation task, and D1–D5 cortical distance in Brodmann area 3b. In Sanchez-Panchuelo et al. (2014), a TW paradigm was also used to measure the somatotopic layout of individual (n = 4) within-digit representations, with S1 Brodmann Area (BA) subdivisions assigned to these maps based on the base-to-tip reversals.

However, phase-encoding methods only provide information on the preferred location of the stimulus on the finger/hand and cannot provide other important measures such as receptive field *size*; the area on the skin represented by an ensemble of neurons. A key advantage of the population Receptive Field (pRF) mapping technique is that it can provide both information of the preferred stimulus location and detail on the extent of stimulus space that drives a particular voxel’s response, including the size and shape (Dumoulin and Wandell 2008; Fracasso et al. 2016; Schellekens et al. 2017). This opens the possibility of comparing results from non-invasive measurements in humans to the results from electrophysiology experiments in non-human primates (NHP). Studies have shown that the size of neuronal receptive fields decreases along the length of the digit, with smaller, more densely packed RFs associated with the digit-tips and higher tactile spatial acuity (Iwamura 1998; Johansson and Vallbo 1979; Mountcastle 2011).

A small number of fMRI studies have reported pRF mapping of the sensorimotor cortex in humans. These include the estimate of pRF size during finger movement using a flexion/extension task (Schellekens et al. 2018), which showed a larger pRF size in the primary motor cortex (M1) compared to the primary somatosensory cortex (S1) (n = 8). This study also demonstrated that movement of the little finger (D5) produced a larger pRF size compared to other digits—an effect which was more evident in the pre-central gyrus compared to post-central gyrus. However, the flexion/extension task did not provide the resolution of “within-digit” maps. A study by Puckett et al. (2019) used somatosensory stimulation of the four digit-tips, with a Bayesian analysis framework to estimate pRF digit maps (n = 6). In that study, a 1D Gaussian profile of spatial tuning across the digit-tips was assumed to estimate location and pRF size of voxels in S1. Schellekens et al. (2021) also stimulated the digit-tips and used a 1D Gaussian model to assess pRF size within Brodmann areas defined from a Freesurfer atlas (Fischl 2012). They showed pRF sizes were smallest in BA3 (rostral wall of the postcentral gyrus), increased slightly towards BA1 (crown of the postcentral gyrus), and were largest in BA2 (caudal wall at base of the postcentral gyrus) (n = 8).

A limitation of these prior fMRI studies is the lack of somatosensory stimulation along the phalanges of the digits. Maps derived from such data can thus only resolve mapping along the medial-lateral (“between-digit”) axis but not in 2D, i.e., also along the proximal-distal (“within-digit”) axis of somatotopic maps. The use of 2D models would make it possible to look for systematic estimation biases, which could occur with limited stimulation-fitting protocols in other previous studies, e.g., differences may arise due to the shape of the underlying pRF rather than size.

A recent 7T fMRI study (Wang et al. 2021) used air-jets for between- and within-digit stimulation of digits D1–D2–D3–D4–D5 at three locations of the tip, base, and palm (15 sites) along the digit (n = 10). Using a 2D Gaussian pRF model and Bayesian inference they showed elliptical pRFs along the digit, with pRFs that were more rounded for D5 and the palm. Their results also suggested larger pRFs in BA1 and BA2 compared to BA3a and BA3b. Another recent fMRI study (Wu et al. 2022) developed a piezoelectric actuated tactile stimulation device for pRF mapping at 3T to stimulate a 5 × 2 grid on the digits (D1–D2–D3–D4–D5) at two within-digit locations (distal phalanx, proximal phalanx). An associated editorial of this work (Gilbert 2022) highlighted the need for more sophisticated stimulation devices for pRF mapping. Additionally, the 3T fMRI study by Wu et al. had a limited spatial resolution for pRF mapping with 3.5 × 3.5 × 4.2 mm^3^ voxels (∼ 51 mm^3^ voxel volume).

Here, we apply passive somatosensory stimulation using piezoelectric stimulators in a 4 × 4 grid with high spatial specificity at four locations along the digits D2–D3–D4–D5 and perform pRF modelling to resolve between-digit and within-digit maps using high spatial resolution fMRI (1.25 mm isotropic voxel size; ∼1.95 mm^3^ voxel volume). We measure pRF size of the cortical digit representations using a 2D Gaussian model (Puckett et al. 2020; Wang et al. 2021), and compare this to a 1D Gaussian between-digit model as used by others (Puckett et al. 2020; Schellekens et al. 2018) and a 1D Gaussian within-digit model (where the Gaussian profile lies along the digits, i.e., orthogonal to the between-digit model).

For each model, we estimate the preferred digit (between-digit direction) and preferred proximal-distal (PD) location (within-digit direction) together with pRF size. We test the following four hypotheses: Hypothesis (1) larger pRF sizes in the within-digit direction compared to the between digit direction; Hypothesis (2) an increase in pRF size moving from D2–D5, Hypothesis (3) larger, more diffuse receptive field coverage maps in BA1 and BA2 compared to BA3b/3a (Puckett et al. 2020; Schellekens et al. 2021; Wang et al. 2021) and Hypothesis (4) a decrease in pRF size moving from the base of the digits to the tips.

## Materials and Methods

Ten participants (7 female, mean age (SD): 28.6 (6.4) years) participated in a 1 h MRI session at 7 Tesla comprising functional and structural scans.

### Vibrotactile Paradigm

**Fig. 1:**
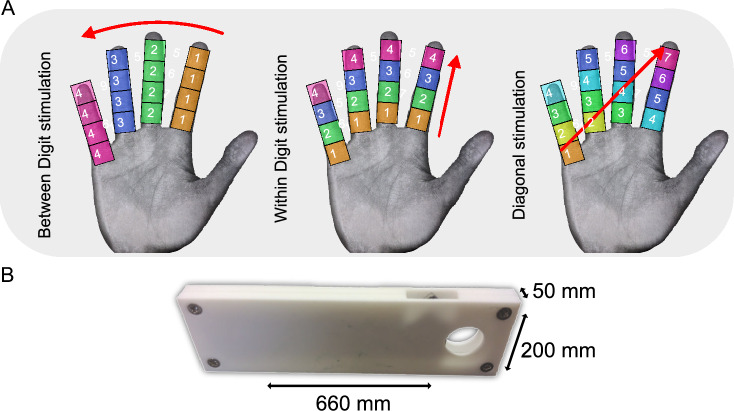
**A** The stimulation protocol. Three stimulation paradigms of ‘between-digit’, ‘within-digit’ and ‘diagonal’ TW stimulation were performed. Numbers indicate which areas were stimulated at the simultaneously. **B** 16 piezoelectric stimulators were placed in a 4x4 grid at four proximal distal (PD) locations along each of the four digits (D2–D5). A single stimulator is shown

Somatosensory (vibrotactile) stimuli were delivered using custom-built, MR-compatible piezo-electric stimulators (Dancer Design, UK; http://www.dancerdesign.co.uk/). 16 independent vibrotactile stimulators were mounted in a non-toxic, reusable modelling compound in a 4 × 4 grid and moulded specifically to each participant’s hand to deliver stimuli with different spatial configurations across digit sites (Fig. 1); each stimulator delivered vibrotactile pulses (30 Hz) to ∼ 1 mm^2^ of skin. An Arduino board (Arduino, Ivrea, Italy; https://www.arduino.cc/), controlled via code written in MATLAB (The Mathworks, NA) and a dedicated amplifier was used to drive the individual stimulators.

In each scan session, three pRF experiments of ‘between-digit’ (BD), ‘within-digit’ (WD), and ‘diagonal’ stimulation were performed (Fig. 1). The ‘between-digit’ and ‘within-digit’ vibrotactile stimulation followed a travelling wave sequence in which 4 locations were simultaneously stimulated in a forward or reverse order in different orientations across the grid. For the ‘between-digit’ and ‘within-digit’ stimulation, 4 s of stimulation ‘ON’ was performed per location (with 4 lines of stimulation resulting in 16 s per cycle) for 12 cycles; for the ‘diagonal’ stimulation, 4 s ON was also performed (with 7 lines of stimulation resulting in 28 s per cycle) for 12 cycles.

### Data Acquisition

MRI data were collected on a 7 T Philips Achieva scanner (Philips, Best, Netherlands) using a 32-channel receive head coil (Nova Medical). Experimental procedures for all studies were approved by the University of Nottingham Medical School’s Ethics Committee. All subjects gave written consent and subjects had no history of neurological disorders. GE-EPI BOLD fMRI data was acquired (TR/TE = 2000/25 ms, field of view (FOV) 144 × 144 × 34 mm^3^ with 1.25 mm isotropic voxels, SENSE factor 2.5 in AP direction, multiband 2 (MB2), 34 slices), both magnitude and phase data were saved. 96 volumes (including a final noise volume) were acquired per run for the ‘between-digit’/‘within-digit’ stimulations, with one/two forward and reverse runs for the ‘between-digit’/‘within-digit’ stimulation. 168 volumes were collected for the diagonal stimulation (as each cycle was 28 s instead of 16 s).

Following the fMRI acquisition, two spin-echo (SE) EPI scans (10 volumes) with matched bandwidth to the GE-EPI were acquired, one with and one without opposing phase encoding, to allow for geometric distortion correction. In addition, structural data were collected using (i) a high-resolution (0.5 × 0.5 × 1.5 mm^3^) T_2_*—weighted FLASH image with the same slice prescription as the EPI data, and (ii) a whole head PSIR T_1_—weighted image with 0.7 mm isotropic resolution.

### Image Pre-processing

Functional MRI data underwent thermal noise removal using NOise Reduction with DIstribution Corrected (NORDIC) PCA (Vizioli et al. 2021) using the magnitude and phase images. This was followed by distortion correction using the SE-EPI data in FSL TOP-UP (FMRIB Software Library), with this correction applied to all GE-EPI fMRI data sets. Using *mrTools* (Gardner et al. 2018) implemented in MATLAB, fMRI data were then motion-corrected and aligned to the high-resolution in-plane T_2_*—weighted scan in the whole-head PSIR space. Data were detrended, high-pass filtered (0.01 Hz cut-off) and converted to percent-signal change for subsequent statistical analyses.

Tissue segmentation and cortical reconstruction of the T_1_—weighted volumes were carried out using Freesurfer (http://surfer.nmr.mgh.harvard.edu/); (Dale et al. 1999). Reconstructed cortical surfaces were flattened in a 55–75 mm radius patch around the S1 hand representation in post-central gyrus, using the mrFlatMesh algorithm (Vista software, https://github.com/vistalab/vistasoft/tree/master/mrAnatomy/mrFlatMesh).

Freesurfer was used to estimate, for each participant, the location of primary and secondary sensorimotor Brodmann areas (BAs 1, 2, 3a, 3b) from a probabilistic atlas based on the histological analysis (Fischl 2012). The maximum-probability map for these was imported into subject-space to define ROIs for each Brodmann area.

### Travelling Wave (TW) Analysis

We first analysed the data using standard Fourier-based travelling wave analysis as a reference to compare our pRF results against. To account for shifts in the fMRI response due to haemodynamic lag, forward and time-reversed reverse scans were appropriately shifted and averaged (Besle et al. 2014). Coherence, phase, and amplitudes of the best-fitting sinusoid were computed. Coherence values were converted to p-values under the assumption of independent Gaussian noise and p-values corrected for multiple comparisons across voxels intersecting the flattened cortical representation, using a stagewise Bonferroni method (Hommel 1988). The phase map was displayed on the flattened cortical patch with a threshold of p < 0.05 (corrected). The corresponding TW data digit-phase response maps (0–2π scale) were binned into 4 colour bands in contiguous π/4 phase bins, with the ‘between-digit’ outputs defining D2–D5 and the ‘within-digit’ outputs defining tip-to-base, termed as proximal-distal locations, PD1 (tip)-PD4 (base).

### Population Receptive Field (pRF) Analysis

Data were analysed subject-wise, by concatenating the ‘between-digit’, ‘within-digit’ and ‘diagonal’ scans for an individual into one time series. Care was taken to consider discontinuities between the end of one scan and the start of the next to ensure no spurious temporal correlations were introduced. To limit the computational time for pRF modelling, a region-of-interest (ROI) encompassing those voxels in the hand region of pre- and post-central gyrus region based on the TW analysis was defined for each dataset (coherence threshold, c > 0.3). This incorporated the post-central gyrus (S1) and additionally, voxels in the pre-central gyrus (motor cortex (M1)).

**Fig. 2:**
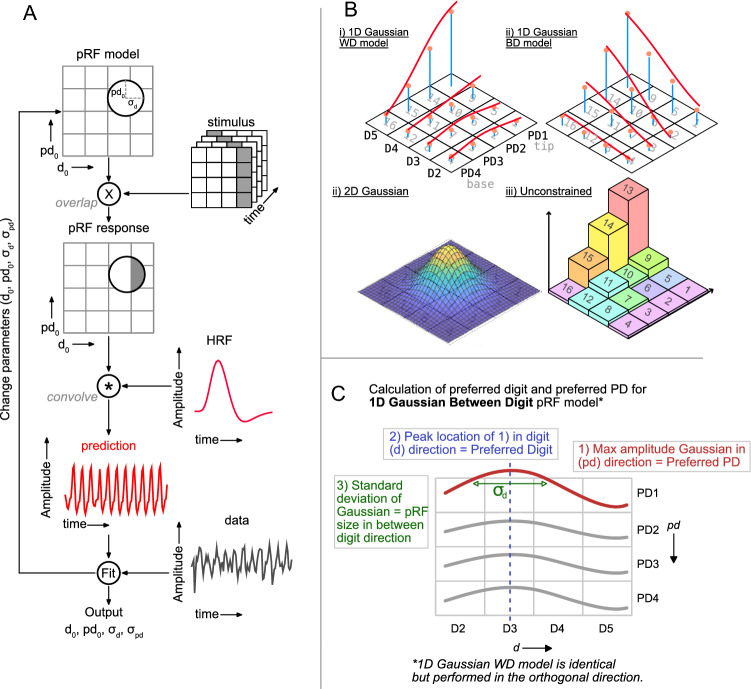
pRF analysis performed on voxel-wise data. **A** To fit a particular voxel timeseries (data, black line), the current estimate of the pRF (weights attached to each location i = 1…16) is combined with the stimulus description and the resulting response estimate convolved with an HRF to account for haemodynamic blurring. To find the best fitting pRF, the model parameters are adjusted via non-linear least squares (Levenberg–Marquardt). This flowchart shows an example for fitting a timeseries with a 2D Gaussian model. **B** Visualization of the different pRF models used. **C** Schematic outlining how the preferred digit, preferred proximal-distal location and pRF size are calculated for the 1D Gaussian BD model. For the 1D Gaussian WD model a similar procedure is performed in the orthogonal direction

For each pRF analysis, a model was generated, e.g., a 2D Gaussian with *d*_*0*_ (along x-axis) and *pd*_*0*_ (along y-axis) defining the pRF centre, and a parameter $\sigma$ defining the spread (where FWHM is ∼ 2.355 $\sigma$). The stimulus description in the somatosensory domain corresponds to a set of matrices which label, for each timepoint, the locations stimulated. The pRF model and stimulus description were combined (dot product) to obtain the predicted pRF response, considered to be the ‘overlap’ between the current estimate of the pRF model and the stimulus description. This predicted pRF response was convolved with a haemodynamic response function (HRF)^1^, to allow for the haemodynamic delay and blurring, resulting in a time series prediction. Next, the residuals between the predicted time series and measured data for each voxel were calculated for non-linear least squares minimization (Gavin 2013). The optimization of these receptive field parameters was taken as the best pRF for the measured voxel (Fig. 2).

The pRF analysis was performed using a modified version of the *mrTools* code(Gardner et al. 2018) originally designed for (visual) population receptive field mapping. To determine the limits of using model fits constrained to the different Gaussian shapes, we compare the 1D and 2D Gaussian models to fits in which each stimulation site is associated with a free parameter weight, imposing no predefined shapes (an ‘unconstrained’ model). We implemented three Gaussian models for pRF descriptions in the somatosensory domain (Fig. 2B), a 2D Gaussian (Model 1), a 1D ‘between-digit’ model (1D Gaussian BD, Model 2) with four independent and separately scaled 1D-Gaussian profiles to describe the response profile with one for each proximal-distal location, while the 1D ‘within-digit’ model (1D Gaussian WD, Model 3) used four independent and separately scaled 1D Gaussian profiles with one for each digit.

Each 1D Gaussian profile was parametrised by the mode/centre (corresponding to the preferred digit-tip) and standard deviation, σ, corresponding to pRF size or tuning width. When fitting the Gaussian, the mode parameter was restricted to the [0.5 4.5] range in fingertip/proximal-distal units (1 = digit 2; 4 = little finger).

The 1D Gaussian BD model *g*(*d*)_*pd*_ combines the four profiles across the digits for each proximal distal (*pd*) location, = 1…4 as follows:

$$g\left( d \right)_{{pd}} = k_{{pd}} \,\,{\text{exp}}\left[ { - \,\frac{{\left( {d_{0} - d_{i} } \right)^{2} }}{{2\sigma _{d}^{2} }}} \right].$$

From this, the Gaussian with the maximum amplitude of the four *pd* locations was used to identify the preferred proximal-distal location, and the location of the peak was used to find the preferred digit along with the associated pRF size ${{\upsigma }}_{d}$, A visualisation of this is shown in Fig. 2C. For the 1D Gaussian fits, the digit or proximal-distal profiles also had a scale factor that allowed their relative amplitudes to be scaled, e.g., for the within-digit model, ${k}_{pd}$ was fixed for d = 2 but allowed to vary for d = 3…5.

The Gaussian 1D WD model, combines the four profiles along each digit *d* = 2…5 along the four locations in the proximal-distal direction:

$$g\left( {pd} \right)_{d} = k_{d} \,\exp \left[ { - \,\frac{{\left( {pd_{0} - pd_{i} } \right)^{2} }}{{2\sigma _{{pd}}^{2} }}} \right],$$

From this, the Gaussian with the maximum amplitude of the four digit locations was used to identify the preferred digit, and the location of the peak was used to find the preferred proximal-distal location and the associated pRF size ${{\upsigma }}_{pd}$. This is effectively an orthogonal version of the between-digit model calculation.

The 2D Gaussian model combines these two spatial terms:

$$g\left(d,pd\right)={k}_{ }\text{e}\text{x}\text{p}\left[-\frac{{\left({d}_{0}-{d}_{i}\right)}^{2}}{2{{\upsigma }}_{pd}^{2}}+\frac{{\left(p{d}_{0}-p{d}_{i}\right)}^{2}}{2{{\upsigma }}_{d}^{2}}\right]$$

where ${d}_{0}$ and $p{d}_{0}$ reflect the location of the pRF peak, ${{\upsigma }}_{d}$ and ${{\upsigma }}_{pd}$ the pRF size along that orientation. Note, in the 1D-between-digit and 2D-Gaussian model, the spatial weighting profile was allowed to cross digits.

To determine the ceiling of the fitted parameters, i.e., to see the maximum possible fitting efficiency of our data to a model without constraints, we also computed fits to an “unconstrained” model (Model 4). For this model, the weights were allowed to vary freely during the fit, with each stimulation site controlled by a simple linear weight. The location of the maximum defined the *preferred digit* (between digits) and *proximal-distal (PD) location* (within digits) but note for this model pRF size cannot be extracted.

For efficient model fitting and to avoid local minima, for each model, the starting values for the parameters were initialised with a grid-search (Dumoulin and Wandell 2008). All pRF outputs were threshold by adjusted r^2^ > 0 (since adjusted r^2^ can be negative). Adjusted r^2^ was used as it penalizes r^2^ by accounting for the number of parameters in the model. The parameters for each model are shown in Supplementary Table 1 (with 2D Gaussian = 9 parameters, each 1D Gaussian = 16 parameters, Unconstrained free parameter = 21 parameters, with all models including 5 HRF parameters). To investigate pRF model performance, the Akaike’s Information Criterion (AIC)(Akaike 1974) which penalizes models with a larger number of parameters, was calculated. For each participant, we calculated the AIC value for all voxels across the times-series. We then calculated the percentage of voxels where a given model had the lowest AIC score, as was performed for the comparison of two visual pRF models in (Kristensen and Sandberg 2021). A matrix of the percentages of lowest AIC scores was then produced to allow comparison across all four models.

### TW and pRF Comparison

To determine the agreement between results of the pRF analysis with the TW analysis, which served as the reference for the pRF analyses, the proportion of voxels assigned to the same digit/PD location by both TW and pRF analysis methods was calculated from the DICE coefficient between the methods. The voxels used for the TW analysis had a coherence threshold > 0.3.

### pRF Size Calculation

To assess the distribution of pRF sizes, the TW ROI was restricted to post-central gyrus only and voxels were aggregated into 16 sites: (i) *functionally*, by the PD stimulation location (PD1–PD2–PD3–PD4) for each of the four digits (calculated from the pRF maps); (ii) *anatomically* by Brodmann area Freesurfer labels (BAs 1, 2, 3a, 3b) for each of the four digits, as assessed in (Schellekens et al. 2021). An anatomical definition of the BAs was preferred to the use of the functional definitions based on the within-digit TW (Fourier) maps because the functional maps are inherently CNR-dependent and thus would not be equally defined across subjects, but BA definitions were checked to confirm correspondence between proximal-distal TW maps and BA maps.

For the 2D Gaussian model, derived values of between-digit pRF size (σ_d_) were plotted against within-digit pRF size (σ_pd_). For the 1D Gaussian models, the 1D Between-digit pRF size σ_d_ was plotted against the 1D Within-digit pRF size σ_pd_, to show whether the pRFs had an elongated shape. The pRF size for each Brodmann area (BA 1, 2, 3a, 3b) and PD location (PD1, PD2, PD3, PD4) was also plotted across all subjects irrespective of digit. For 8/10 subjects who had significant activation of pre-central gyrus, a similar analysis was performed restricted to Brodmann areas 4a, 4p and 6. Single-factor ANOVAs were performed to identify any differences in pRF size between Brodmann areas, proximal distal locations, or digits, and multiple comparisons was performed using Tukey’s HSD (*multcompare*() in MATLAB). The volume assigned to each of the 16 Digit/BA sites was also computed, and significant differences determined.

### pRF Coverage Map Calculation

For the 1D and 2D Gaussian models, pRF coverage maps were generated to visualize the representative pRF shape in each Brodmann area ROIs x Digit ROIs (16 ROIs). This was performed for 10 participants for S1 (BA 1, 2, 3a, 3b) and 8 participants for M1 (BA 4a, 4p and 6).

To do this, we first extracted the optimised fit parameters from every voxel in each subject. For each voxel within these 16 ROIs, there was a pRF grid comprising a 4 × 4 matrix, which contained the weights of the pRFs for all voxels fit. Next, we interpolated each voxel in the pRF 4 × 4 grid to a 20 × 20 grid; following a similar method to that performed by others, for example (Puckett et al. 2020) used a 10 × 10 grid and (Wang et al. 2021) used a 12 × 12 grid, and aids visualisation of the pRF field maps. To visualise the “missing” part of the pRF coverage maps, the grid was then extrapolated to 60 × 60, filling in the gaps around the 20 × 20 grid by solving a partial differential equation to recover the ‘full’ pRF shape (D’Errico 2012).

To identify the most common pattern across voxels within each of the 16 ROIs, we then performed principal component analysis (PCA) in MATLAB (*pca*()) to identify the “patterns” that explained the greatest variance. We took the first 3 principal components (that explained ∼ 90% of the variance) and weighted the principal components subject-wise for each of the 16 ROIs to generate a resultant *average pRF coverage map* across subjects; this weighting method eliminates possible bias from few subjects dominating pRF patterns. We averaged these pRFs (1) across Brodmann areas and (2) across digits. Further, to derive an average coverage map, the peaks of each location specific pRF were aligned in the digit direction, and the proximal-distal direction separately (Fig. 8). This procedure ensures that the summary captures the average pRF shape of digits regardless of the exact location in stimulus space.

In addition to computing the pRF coverage maps in S1 by Brodmann area, they were also calculated by proximal-distal (PD) location ROIs x Digit ROIs (16 ROIs).

## Results

### Preferred-Digit Maps, Proximal-Distal Maps, and Brodmann Areas

**Fig. 3:**
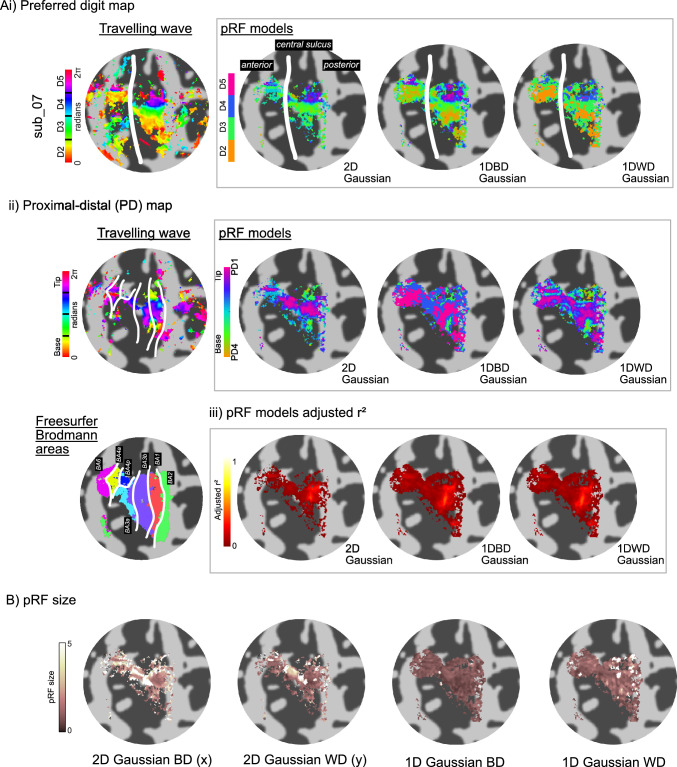
**A** Travelling wave fourier analysis and pRF model fit maps for 2D Gaussian and 1D between-digit (BD) and 1D within-digit (WD) Gaussian shown for an example subject (#7). pRF fit maps are threshold at an adjusted r^2^ > 0. (i)  Preferred digit maps from the between digit stimulation, showing the TW map and four pRF model outputs. Digits can be seen to move from inferior (D2) to superior (D5). (ii) Preferred proximal-distal maps from within-digit stimulation. Tip-base stimulation results in phase reversals, suggestive of functional separation of Brodmann areas. Also, shown are the anatomical Freesurfer labels of Brodmann areas. (iii) Adjusted r^2^ maps for each pRF model. **B** pRF size defined as σ, note that two σ maps are output for the 2D Gaussian model, for x and y directions, respectively

Distinct *preferred-digit* and *preferred-proximal-distal* (PD) maps were apparent for all subjects. Figure 3 shows, for an example subject, the preferred-digit, and preferred-proximal-distal maps from the TW and pRF analysis, together with maps of adjusted r^2^, and pRF size. The preferred-digit maps show clear digit representations from D2 to D5 (orange to pink from the bottom of the flat map towards the top), while the preferred-proximal-distal maps show reversals of base to tip locations corresponding to functional areal borders between mirrored representations in BA3a, 3b, 1, 2 (Sanchez-Panchuelo et al. 2012). Supplementary Fig. 1 shows the TW and pRF output maps for each of the 10 participants.

The relative volumes of the digit ROIs (calculated from number of voxels × voxel volume) separated by Brodmann areas are shown in Supplementary Fig. 3. On separation of digits by BAs, in the post-central gyrus, the volume of BA3a was found to be smaller than BA1 and BA3b (*t *test, n = 10 subjects: BA3a < BA3b p = 0.004, BA3a < BA1 p = 0.005), while in the precentral gyrus BA4p was greater than BA6 (BA4 p > BA6 p = 0.01). For the digits, within the postcentral gyrus the volume of D2 and D3 were larger than D5 (D2 > D5 p = 0.016, D3 > D5 p = 0.027), and in precentral gyrus D2 was greater than D4 and D5 (D2 > D4 p = 0.048, D2 > D5 p = 0.015).

### pRF Model Assessment and Comparison with Travelling Wave

**Fig. 4:**
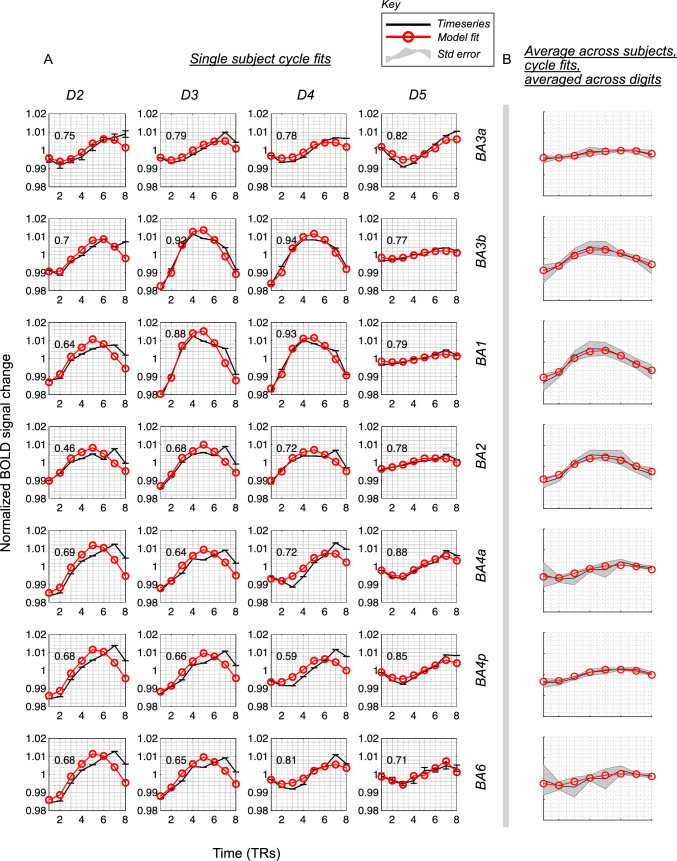
**A** The single cycle timecourses and their fits (red line) from the 2D Gaussian model from an exemplary voxel in an individual subject (Subject 7). **B** Single cycle fits are shown averaged across n = 8 subjects (to include all subjects which show responses also in BA4a, 4p, 6). Error bars are standard error across subjects. Data shown for one set of forward scan cycles for each digit and average across digits

Figure 4 shows model fits for a single cycle of stimulation, split across Brodmann areas and digit for an individual subject (A). Cycle fits averaged across the average of n = 8 subjects are shown in (B). Data are shown from the 2D Gaussian pRF model fits which can be seen to fit well.

Supplementary Fig. 4 shows example model fits in an individual subject across the entire timeseries, showing those voxels with both good and poor fitting voxels.

**Fig. 5:**
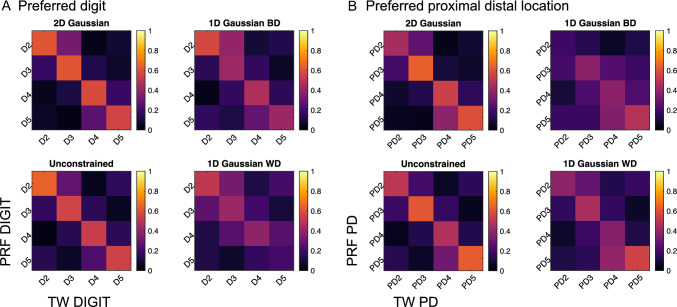
Dice coefficient comparing the TW to the pRF analysis for **A** preferred digit and **B** preferred proximal-distal (PD) location for the 2D Gaussian and. Measures are computed for data threshold at an adjusted r^2^ > 0, the dice score shows the mean across all subjects

First, to assess the pRF models, the maps of preferred-digit and proximal-distal location were compared to the TW analysis, Fig. 5 shows the dice coefficient between the pRF analysis and TW analysis of the (A) preferred digit and (B) preferred proximal distal location (results are the average across all participants). Good agreement is seen in the preferred-digit and proximal-distal location across all pRF models, as indicated by the higher dice values along the diagonal. The 2D Gaussian can be seen to result in higher Dice scores, matching most closely the unconstrained fit which is indicative of maximum Dice coefficients.

**Fig. 6:**
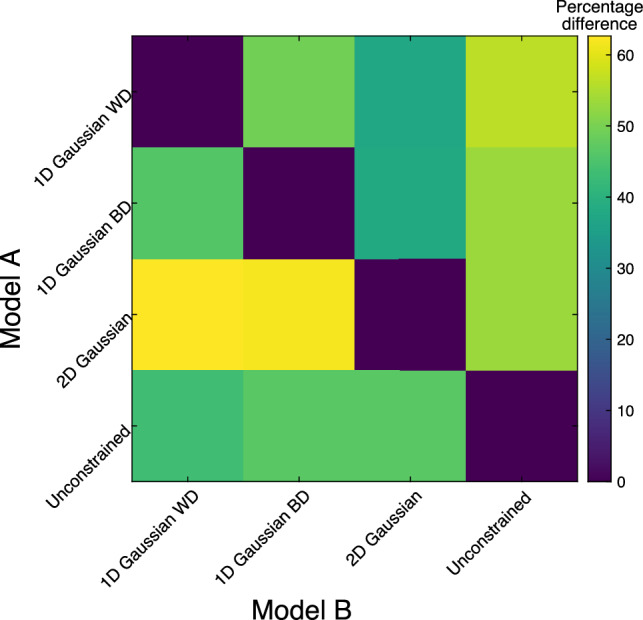
The percentage differences of AIC scores between models. Each model's AIC was compared to every other model and the percentage of lower AIC scores in model A vs. model B is shown as a matrix. The y-axis is compared to the x-axis, such that, e.g., for the 2D Gaussian model vs. the 1D Gaussian Within Digit model, ∼ 65% of voxels have a lower AIC for the 2D model. A higher percentage indicates a better model for model A vs. model B

To compare pRF model fits, the percentage difference in Akaike Information Criterion (AIC) between models is shown in Fig. 6 (higher percentage difference between Model A vs. Model B = better for Model A). The 2D Gaussian had the lowest AIC scores, although this did not reach the criteria of being significantly lower (p < 0.001) than other models.

### Investigating pRF Size

**Fig. 7:**
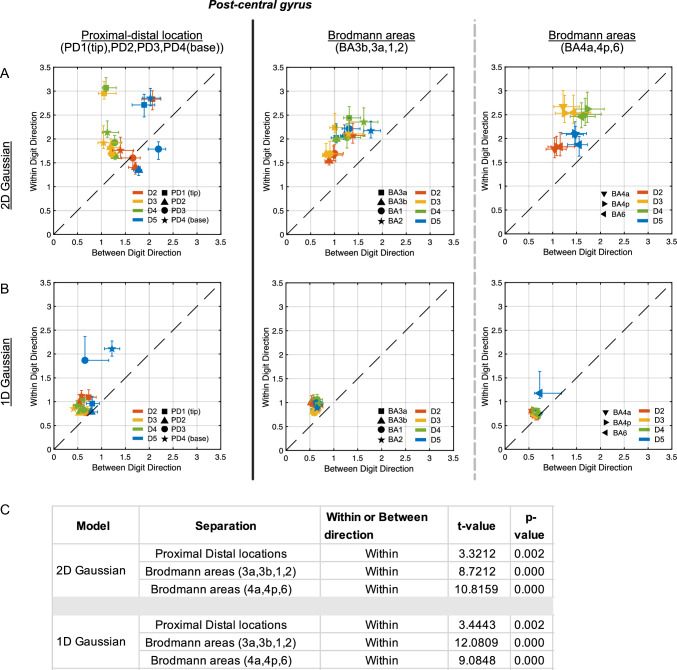
pRF size computed in the between digit (σ_d_ = x) and within digit (σ_pd_ = y) direction for the **A** 2D Gaussian model and **B** 1D Gaussian model. Data are displayed for somatosensory cortex (S1) by dividing the data into base-tips (proximal-distal (PD) locations) and Brodmann areas (BA). Values displaced from the line of identity y = x  indicate a preference for elliptical pRF shapes, compared to circular pRFs along the line of identity. The units on the axes are arbitrary and relate to the pRF size measured in grid units (4 × 4). The different colours represent digits, and the shapes represent Brodmann areas. Each point is a group average (n = 10) for the colour, e.g., D2 and shape, e.g., BA3a, which would be a red square. Responses in the precentral gyrus from Brodmann areas 4a, 4p, and 6 could be measured in 8 of 10 subjects. **C** Paired *t *test of pRF size in the between and within digit direction showing any significant preference for direction

Figure 7 shows the group average pRF size for the 2D Gaussian model (of within-digit σ_pd_versus between-digit σ_d_), the 1D Gaussian WD (σ_pd_) and 1D Gaussian BD model (σ_d_), separated by functional proximal-distal (PD, base-tip) location or anatomical Brodmann area (BA) for each digit. There is a strong and consistent preference for larger pRF size in the within-digit direction than between-digit suggesting an elongated pRF shape (pRF sizes along the line of identity *x* = *y* would be a perfectly circular shape) as predicted for Hypothesis 1.

In Fig. 7, when comparing pRF size across digits, the 1D Gaussian WD and 1D Gaussian BD models showed larger pRF sizes for D5 compared to the other digits (1D Gaussian WD: D5 > D2 p = 0.003, D5 > D3 p < 0.001, D5 > D4 p < 0.001; 1D Gaussian BD: D5 > D2 p < 0.001, D5 > D3 p < 0.001, D5 > D4 p < 0.001). This difference of pRF size across digits was only significant when splitting the 1D Gaussian response maps by PD location, and not when splitting pRF size by Brodmann area. The 2D Gaussian showed differences in digits, with D4 > D2, when separating digits by Brodmann areas (p = 0.037), in the within-digit direction (y-axis in Fig. 7), and D5 > D3 p = 0.014, D4 > D3 p = 0.028, in the between-digit direction (x-axis in Fig. 7). When separating digits by PD, in the between digit direction, D5 had larger pRF sizes (D5 > D3 p < 0.001, D5 > D4 p < 0.001). D2 also had larger pRF sizes when compared to D3 (p < 0.001) and D4 (p < 0.001). Supplementary Table 2B details these significant differences in pRF size for the 2D and 1D Gaussian models, and Supplementary Figs. 5 and 6. These results support Hypothesis 2 of an increase in pRF size moving from D2–D5.

**Fig. 8:**
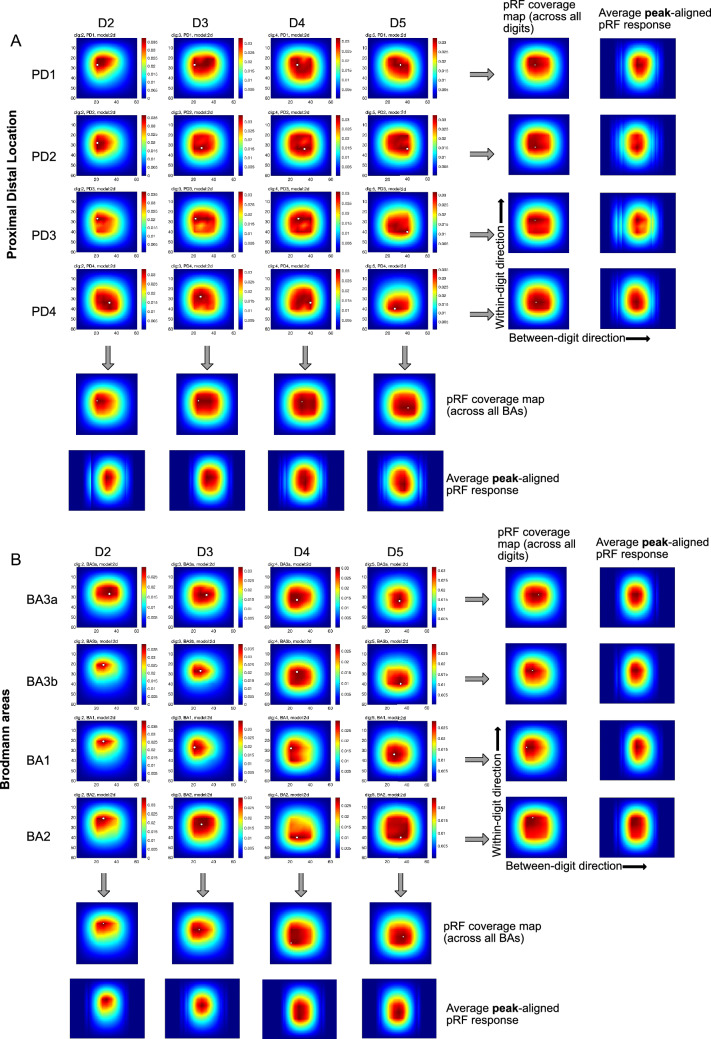
pRF coverage maps from PCA analysis for the 2D Gaussian model shown for **A** Proximal Distal location and **B** Brodmann Area. The pRF coverage map refers to the averaged area of influence that the ROI set of voxels exerts in sensory space The pattern shows a column of preferred responses moving from left to right, indicating each digit. **A** The PD location shows a more spread pattern from tip to base PD1–PD2–PD3–PD4. **B** shows a more spread pattern for Brodmann areas BA3b–BA1–BA2. As the digit centres are not aligned, simply averaging the coverage maps across digits produces a blurred representation (pRF coverage map across digits). To correct for this blurring in the x-axis, we also computed the peak-aligned average. The white dot indicates the peak value on the grid. Data shown in this figure is restricted to a somatosensory cortex (post-central gyrus) ROI. We also peak aligned across BA or PD areas

There were significant differences in pRF size when separating across Brodmann areas in the between digit direction. BA2 was larger than BA3b for 1D Gaussian BD model (p = 0.021), and larger than BA3a, 3b and 1 for the 2D Gaussian model in the between digit direction (p < 0.001), supporting Hypothesis 3. There were also significant differences in pRF size across the proximal-digit (PD) location, with some support for the base tending to have a larger pRF size (Hypothesis 4). PD4 was greater than PD2 in 1D Gaussian models (p = 0.008), in the within-digit direction and greater than PD2 (p < 0.001) and PD3 (p = 0.021) for the 2D Gaussian model. The 2D Gaussian model had greater pRF size in PD1 (tips) than PD2, PD3 and PD4 (base) (p < 0.001), but also greater pRF size of PD4 than both PD2 and PD3, in the within-digit direction. See Fig. 8 and Supplementary Table 2A.

To visualise the coverage of sensory space by population receptive fields, the average pRF coverage map across subjects is shown in Fig. 8 for the 2D Gaussian model separated by (A) PD location and (B) Brodmann Area. The 4 × 4 grid shows a preference for digit selectivity (D2–D3–D4–D5) from left to right, with the response pattern seen to be most focal for D2 and D3. The PD location coverage maps (Fig. 8A), show the shift in peak location from D2–D3–D4–D5 (in x-direction) as well as from tip to base (PD1–PD2–PD3–to PD4 in y-direction). When digit-aligning the peaks, the average peak-aligned pRF response tends to show that the digit tip (PD1) is smaller than the digit base (PD4) (Fig. 8A) (Hypothesis 4). Similarly, the BA coverage maps (Fig. 8B), show a tendency for a shift in peak location from D2–D3–D4–D5 (in x-direction) whilst the average peak-aligned pRF response show BA1 and BA2 appear visually larger and more diffuse than BA3a and BA3b (Hypothesis 3).

Supplementary Fig. 4 visualises the pRF coverage maps for the 1D Gaussian BD and WD models. For the 1D Gaussian models, the pRF coverage maps show a more focal response compared to the 2D Gaussian model; this is more evident in the proximal-distal than Brodmann area separation. Compared to the 2D Gaussian model, it is easier to see a shift in peak locations from PD1 (tip) to PD4 (base), and D2 to D5, however, there is little change in shape or diffuseness that is consistent with our hypotheses. Notably, the 1D Gaussian BD model shows an increase in size from D2–D5 (Hypothesis 2). Supplementary Figs. 5 and 6 show pRF size across digits, summed across PD location and BA respectively, together with the associated 1D and 2D peak-aligned pRF coverage maps for the digits.

**Fig. 9:**
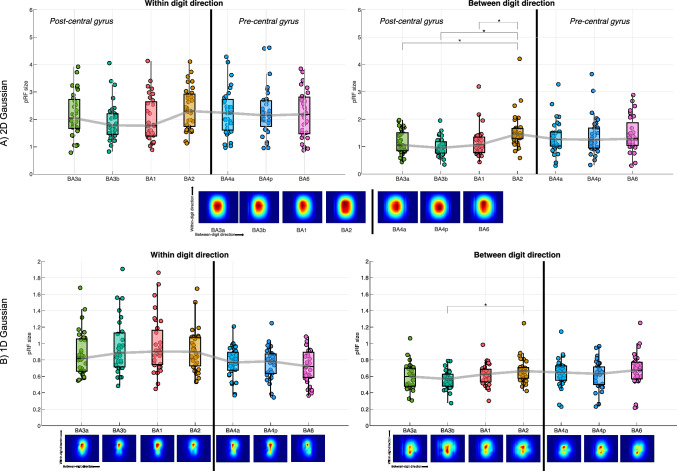
pRF size collapsed across digit and shown for each Brodmann area. For BA 3a, 3b, 1 and 2, responses were restricted to somatosensory cortex (post-central gyrus, n = 10) and for BA 4a, 4p, 6 to the motor cortex (pre-central gyrus, n = 8). The grey line connects the median of each box-whisker chart. Asterisks indicate significant differences (p < 0.05). Each point on the box-whisker charts is the mean from an ROI for a given subject.  The pRF coverage maps are shown beneath for each model. **A** 2D Gaussian model with within-digit and between-digit results shown, and  **B** 1D Gaussian within-digit and 1D Gaussian between-digit models

Figure 9 summarises the result of pRF size across Brodmann area, showing the 1D and 2D models results in Fig. 7 summed across digits. In addition, we show the associated 1D and 2D peak-aligned pRF maps, for which the pRFs were centered across digits before averaging, to provide a more accurate picture of pRF shape, regardless of the exact position in sensory space. Larger pRF sizes are seen for BA2 in all models, but this is most clear for the 2D Gaussian model.

**Fig. 10:**
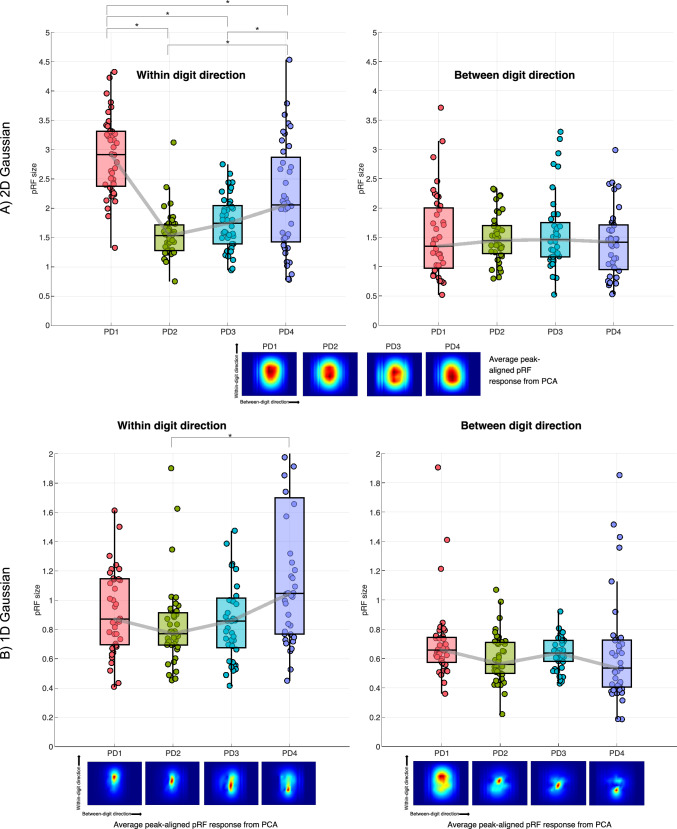
pRF size collapsed across digit and shown for proximal-distal location (PD1 (tip) to PD4 (base)) in somatosensory cortex (post-central gyrus, n = 10). The grey line connects the median of each box and whisker chart. Asterisks indicate significant differences (p < 0.05). Each point on the box-whisker charts is the mean from an ROI for a given subject.  The pRF coverage maps are shown beneath for each model. **A** 2D Gaussian model with within-digit and between-digit results shown, and **B** 1D Gaussian within-digit and 1D Gaussian between-digit models

Figure 10 shows the result of the pRF size summed across PD location in somatosensory cortex (S1) for the 1D and 2D Gaussian models. Again, pRFs were aligned both across digits and PD location to provide a clearer picture of pRF shape (see also Fig. 9). Larger pRF sizes are seen for PD4, again this is most clear for the 2D Gaussian and can also be seen in the 1D Gaussian WD model.

## Discussion

We performed pRF mapping of the human somatosensory cortex using a closely spaced 4 × 4 piezoelectric stimulation grid to generate a 2D pattern along the phalanges and across digits with reproducible localization. We measured pRF responses using high resolution, 1.25 mm isotropic fMRI data corrected for thermal noise with NORDIC. We compared a 2D Gaussian pRF model of the stimulation grid, with a more standard 1D between-digit Gaussian model (Puckett et al. 2019; Schellekens et al. 2021), 1D within-digit Gaussian, and an unconstrained model.

### Comparison of Preferred-Digit and Preferred Proximal-Distal Location PRF Maps with Ground Truth Fourier-Based TW Derived Maps

Results of both the Fourier-based TW analysis and pRF models showed an orderly representation between digits (Besle et al. 2013; Kolasinski et al. 2016; Martuzzi et al. 2014; O’Neill et al. 2020; Schweisfurth et al. 2015), with clear within-digit reversals from tip-base (Sanchez-Panchuelo et al. 2012). To quantify the similarity between the maps derived with the different techniques, we calculated dice coefficients. Comparison of pRF derived maps with TW maps indicate all models did well in matching their assignments of preferred digit and proximal-distal location (Fig. 3). At the adjusted r^2^ threshold, the 2D Gaussian maps were spatially sparser than the Fourier-based TW map (threshold at coherence > 0.3) whilst dice scores were poorer for 1D Gaussian models than the 2D Gaussian and unconstrained models.

### Proximal-Distal Maps are More Complex than Preferred Digit Maps

Here we used a 4 × 4 grid providing sufficient stimulation sites to separate both within-digit (proximal-distal, PD) and between-digit maps. It is important to note that within-digit proximal-distal maps are not expected to be simply a 90-degree rotated version of the between-digit maps on the cortical surface. Preferred digit maps show a smooth gradation in cortical space. Proximal-distal location maps, in contrast, contain maps which are mirror-reflected at boundaries between Brodmann areas 3a, 3b, 1 and 2 across a single digit (Sanchez-Panchuelo et al. 2014).

To capture both between and within digit assessment simultaneously, we applied a 2D formulation of the pRF model which provides voxel-wise quantification of preferred stimulus location as well as pRF size in the between and within digit orientation. To summarise and compare the results of pRF mapping, we created regions of interest by dividing S1 in two ways, either by proximal-distal location (PD1–PD2–PD3–PD4) or Brodmann areas ROIs (BA1, 2, 3a, 3b). In addition, we also assessed Brodmann Areas 4a, 4p, and 6 in the pre-central gyrus motor cortex (M1).

### pRF Size and Comparison with Previous Studies

Previous studies using pRF mapping in somatosensory cortex have mostly stimulated digit-tips (Puckett et al. 2019); Schellekens et al. 2021) and used a 1D Gaussian BD model. Using a 1D Gaussian BD model (Schellekens et al. 2018) showed the Gaussian spread depended on the estimated preferred finger digit, with larger pRFs for D5, the little finger, compared to relatively small pRFs for the thumb (D1). However, they found no clear trends when looking at pairwise differences between D2–D4. In line with the previous imaging and behavioural literature, we tested four hypotheses: (1) pRF sizes in the within-digit direction would be larger than in the between-digit direction; (2) pRF sizes increase from D2 to D5; (3) pRF receptive field coverage maps would show a more diffuse pattern in BA1/2 compared to BA3b/3a and 4) decrease in size from the base of the digits to the tips.

Our results showed the average pRF size was significantly greater in D5 compared to D2, D3, and D4 using both 1D Gaussian models (Hypothesis 2). This was more clearly depicted in the 1D Gaussian BD model than 1D Gaussian WD model (Supplementary Figs. 4, 5 and 6) when separated by PD. Interestingly, for the 2D Gaussian model formulation the significant differences in pRF size across digits D2–D5 was more clearly seen for separation by BA than PD but evident in pRF coverage maps of both (Fig. 8, Supplementary Figs. 5 and 6). It is noted that each axis of the 2D Gaussian model is not independent, and therefore looking at each separately does not represent the coverage map (shape) well, unlike for the 1D models, where each axis is independently modelled. Previous behavioural results mostly indicate a declining sensory sensitivity from D2 to D5. For example, some studies investigating point localization and two-point discrimination, have shown a better performance for thumb, D2, D3 as compared to D4 and D5 (Manser-Smith et al. 2018). D5 has also been shown to have the worst localization threshold (Louis et al. 1984; Schweizer 2000), with similar patterns found in active and passive volume perception (Zhang et al. 2018, 2019). In addition, in studies investigating spatial acuity, a lower spatial acuity has been shown for D5 as compared to D2–D4 (Duncan and Boynton 2007; Sathian and Zangaladze 1996).

The 2D Gaussian model resulted in larger and more variable pRF size estimates than the 1D models (Fig. 7). For both 1D and 2D Gaussian models, we show increased pRF size within-digits compared to between-digits leading to elongated pRF estimates, as shown in Fig. 7, and visualised in the average peak-aligned pRF responses in Fig. 8 (Hypothesis 1). A recent study (Wang et al. 2021) using somatosensory stimuli delivered by air-jets also reported more elongated pRF shapes in the within-digit orientation, they also showed more circularly symmetric pRF shapes for D5 and the palm.

Grouping pRF size results based on Brodmann areas or PD location makes a difference, particularly because of the divergent pRF size estimates for the tip.

Across Brodmann areas, there were larger pRF sizes in BA2 compared to BA3b (seen for the 2D Gaussian and 1D Gaussian BD models), consistent with electrophysiological literature and Hypothesis 3. PRF sizes in area BA2 were larger than in BA1. Brodmann areas in the pre-central gyrus (BA4a, 4p and 6) had pRF sizes comparable to that of BA2 (Fig. 9).

On separating by PD location, pRF sizes tended to be largest for the digit base (PD4) (Hypothesis 4**)** but this dominance was digit dependent, and this trend was inconsistent across all pRF models, with the 2D Gaussian pRF model showing larger pRF sizes for the tips. It can be seen from the coverage maps the shape of the pRFs across PD (Fig. 10).

The 2D Gaussian model provided the most physiologically relevant pRF coverage maps—a mixture of 2D Gaussians, indicating the shape and spread of the receptive fields in aggregate (Fig. 8). However, it should be noted that 1D models provided visually consistent pRF coverage maps along their respective dimension (Supplementary Fig. 4). The 1D Gaussian WD case is likely more ecologically valid than a 1D Gaussian between digits version, which imposes a smooth Gaussian shape in the sensory space across digits.

### Comparison of Models Fits

Visually, the 1D Gaussian models provided digit maps that more closely represent the fit ceiling of the unconstrained model and the TW Fourier maps, than the 2D Gaussian model. We suggest that this occurs due to a hierarchical reduction in parameters moving from the 1D to the 2D model, i.e., the spatial constraints inherent in the 2D Gaussian model formulation gave rise to lower r^2^ values compared to other models, especially the unconstrained models which has the largest number of free parameters. To assess the optimal model, we assessed which models had the lowest AIC scores by computing the percentage of voxels with lower scores for a given model; this was shown to support a better model fit for the 2D case, though this did not reach significance. The pRF size difference between models likely relates to the fact that the 2D model likely provides a more precise estimation of digit pRFs. Therefore, this model may be better at highlighting differences that would go undetected in 1D models.

### Limitations

Compared to visual cortex, where pRF methods were developed and have been widely used, the setup of stimulation sites is far coarser in somatosensory space. This is a limit imposed by the stimulation delivery devices. Here, the goal was to stimulate using precise piezoelectric stimulators with a denser array than had previously been used in studies. This coarser resolution limits the choice of pRF models that can be used without regularisation or other approaches to deal with underdetermined problems. Using pRF shapes based on a Gaussian distribution provides good fits for visual cortex data (Dumoulin and Wandell 2008). In addition, elaborations of this model using the difference of Gaussians, adding static non-linearities and even multi-stage cascade models (Kay et al. 2013) have been shown to work even better. Here, we assessed the 1D Gaussian BD model and 1D Gaussian WD models. The simple 1D Gaussian WD model takes into the account the discontinuities between the digits, while preserving the continuous set of receptive fields within the digit.

Attempts at regularising the pRF fit were limited by the coarseness of our stimulator grid (Lee et al. 2013). In future studies, more stimulators could be added to the digit surface, although this may be practically limited by stimulator size and coupling between stimulators, causing a blurring of sensation, especially within the digit, i.e., it may become harder for the subject to identify where along the digit is being stimulated, and hence this top-down “confusion” may affect sensory maps, which have been shown in previous work to have an attentional component (Puckett et al. 2017).

Previous literature has stimulated the thumb (e.g., (Schweisfurth et al. 2014), however, due to our current limit on stimulator numbers (16), in this study the focus was on D2–D5. We used a non-toxic, reusable modelling compound to create a malleable frame for a subject’s hand to rest comfortably on the 16 stimulators. Other placement arrangements were explored including a bespoke glove, however, due to the size of the stimulators and the fact that they had to be placed lengthwise (increasing the area occupied by them in the glove), this proved to be non-optimal.

The pre-central cortex contribution in our ROIs was small compared to the post-central cortex, and responses were seen only in 8/10 subjects. Given that areas 4a, 4p and 6 are within the motor cortex, it may be that responses observed here may be due to movement of the fingers during somatosensory stimulation. In future work, one could perform a pure somatosensory task (as performed here), followed by a motor task (as performed in Schellekens et al. 2018 in the same subject to assess whether pRF size differs between motor and passive stimulation tasks.

In this study, a TW paradigm was used with three axes of movement (between and within digit, and diagonal). It is possible that the effect of a known TW stimulation pattern may have influenced the estimated pRF shape and size, along with the effects of attention/cueing. Prior studies have looked at comparing “travelling wave” with “event-related” designs comprising trials in a randomised order (e.g., (Besle et al. 2013) from our lab) and have shown both designs yield very similar fingertip-specific maps, while the effect of attention has been shown to be modulated at the level of the cortical representation of individual digit responses (e.g., (Puckett et al. 2017). To minimise such effects, in this study the direction of the TW stimulation was varied (for the between and within digit conditions) and the design included a diagonal stimulation, which creates a pattern of sensation on the hand that participants found much harder to predict. Future studies should compare a TW design with a fully randomised design to assess whether this results in more unbiased pRF estimations.

## Conclusion

Here, we use a novel 16 stimulator setup (a 4 × 4 grid) to measure two-dimensional sensory maps of between-digits and within-digits (D2-D4) sites using population receptive field (pRF) mapping with high spatial resolution fMRI data obtained at 7T. Using 1D and 2D Gaussian pRF models, we estimated pRF size and shape in S1 and M1 for each digit and compared data by aggregating voxels in regions of interest corresponding to within-digit (tip-to-base) location and by anatomically defined Brodmann areas. We also compared these results to those obtained with 1D Gaussian models.

Consistently across 1D and 2D Gaussian models, we found elongated pRF shapes along (within) the digits, and a general increase in pRF size moving from D2–D5, across BA3b-2, and across PD locations from PD1 (tip) to PD4 (base). Future studies would benefit from a denser stimulator setup to study 2D pRF mapping that can be used easily across multiple subjects.

### Supplementary Information

Below is the link to the electronic supplementary material.

Supplementary material 1 (DOCX 27805.9 kb)

## Data Availability

All code used in this article has been developed in MATLAB and is hosted on a public GitHub repository (https://github.com/ppxma7/pRF_somato_data). Processed data to reproduce the analysis are hosted on a Mendeley data repository (10.17632/h2sdnt8223.1). The raw data can be shared on request.

## Footnotes

1 The shape of the HRF was assumed to be “canonical”, although voxel-wise HRF estimates can also be obtained.
