# Supplementary material for: Two-Dimensional Population Receptive Field Mapping of Human Primary Somatosensory Cortex
Source: Brain Topogr. 2023 Aug 27;36(6):816–34. doi: 10.1007/s10548-023-01000-8 (PMC10522535; doi:10.1007/s10548-023-01000-8)
Supplement: Supplementary file 1 — Supplementary material 1 (DOCX 27805.9 kb) [file 10548_2023_1000_MOESM1_ESM.docx]

**Supplementary Information**

**Supplementary Figure 1:** Travelling Wave (TW) between digit (BD) and within digit (WD) maps, and population receptive field (PRF) BD and WD maps shown for all subjects (n=10). Subject 7 (outlined) is shown in the main text.

**Supplementary Figure 2:** Example time series using the same 2 voxels (one that fits well, and one that fits poorly), fit using each of the 4 models. The mean and standard errors of the timeseries are shown on the right of each timeseries plot. Data are shown for a single subject.


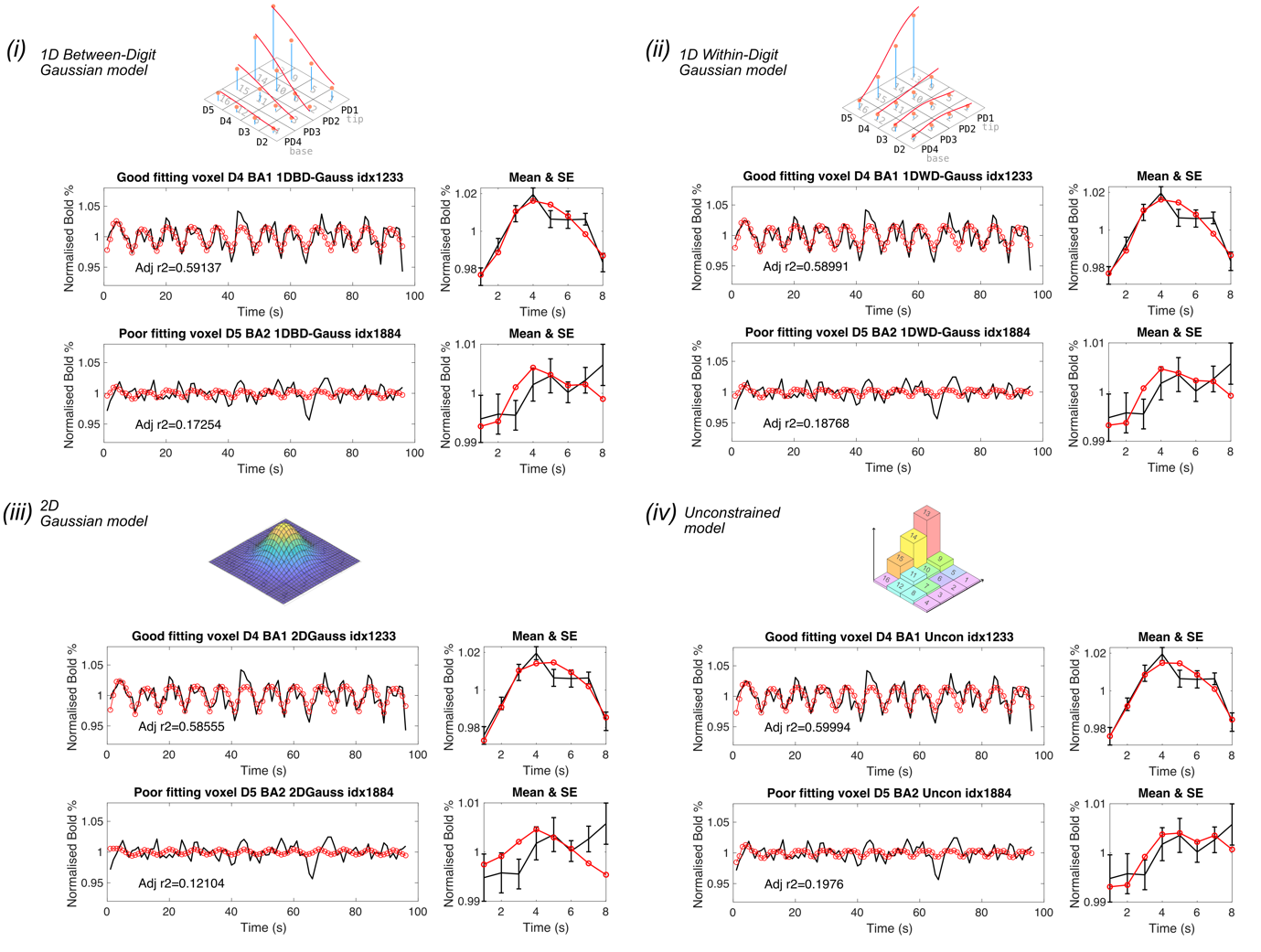


**Supplementary Figure 3:** Volumes of digit and Brodmann area (BA) ROIs, with average and standard deviation across subjects shown. BA 3a, 3b, 1 and 2 are shown for n=10 subjects, while BA 4a,4p,6 areas are shown for a smaller n=8 subject pool. A) Grouped by BA and coloured by digit. B) Grouped by digit and coloured by BA. Asterisks indicate significance as shown in C) ANOVA performed on ROI volumes, with BA and digit as factors. Significant differences that survive multiple comparisons correction are tabulated (p<0.05).


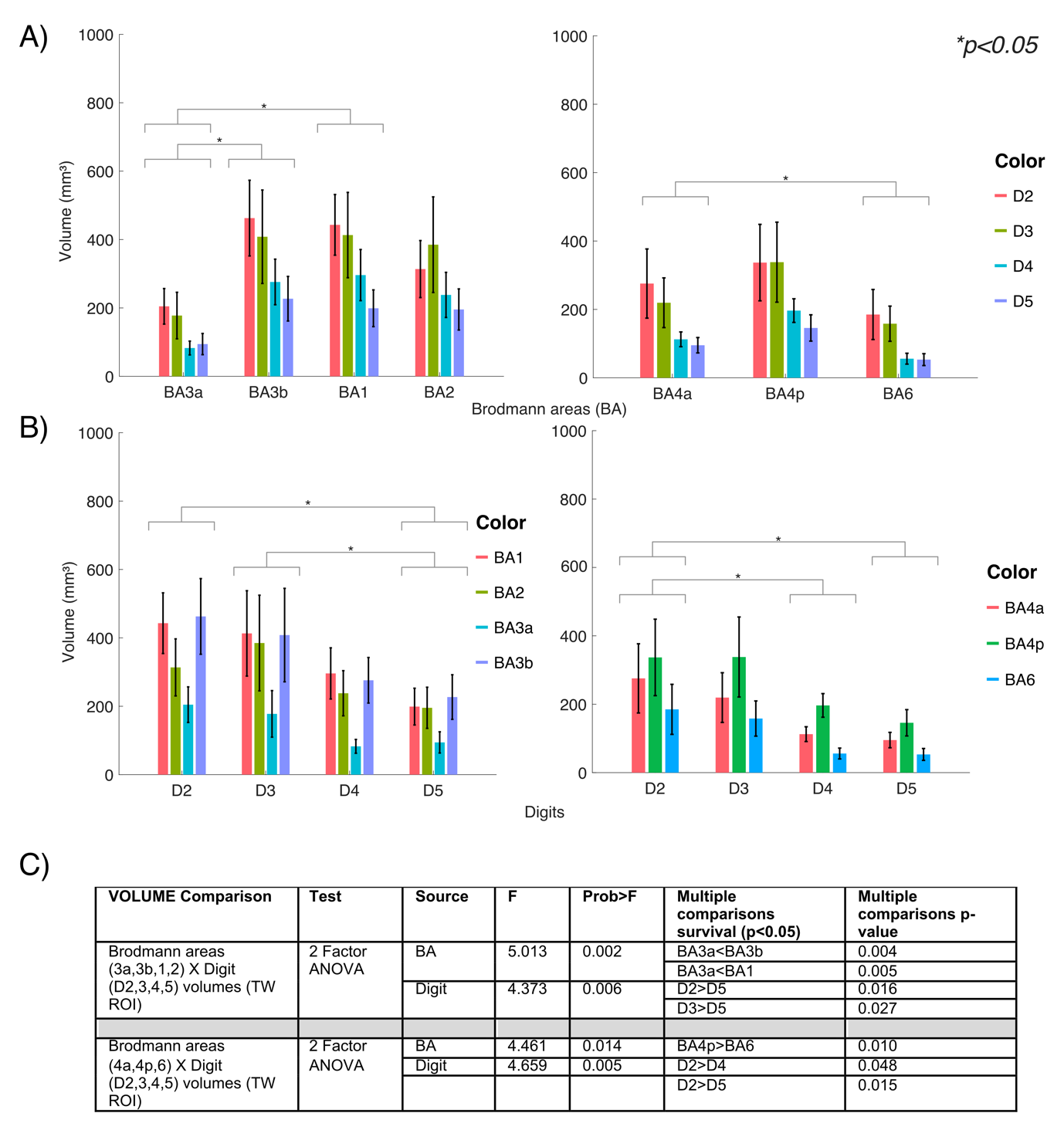


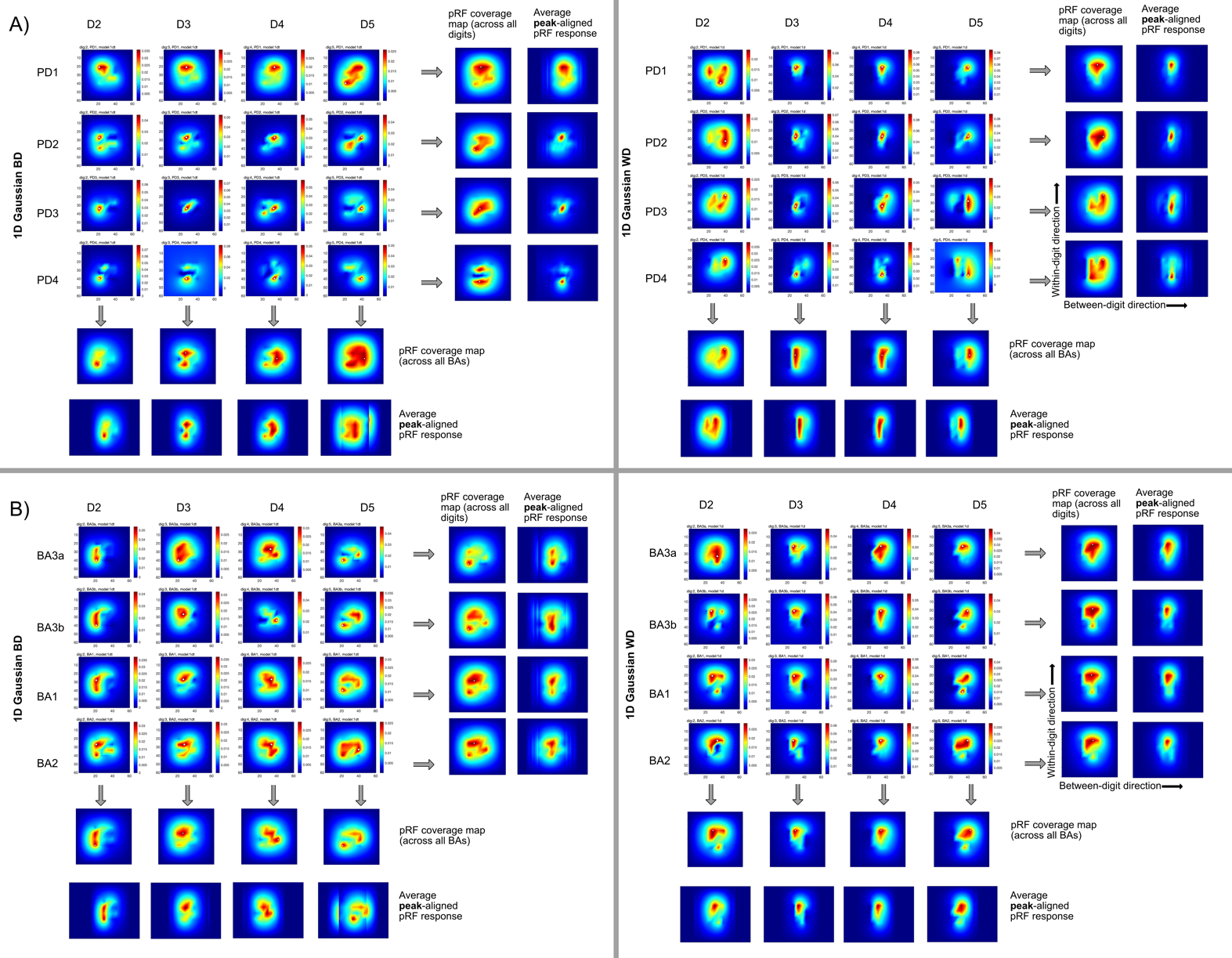
**Supplementary Figure 4:** pRF shapes from PCA analysis for the 1D Gaussian WD and 1D Gaussian BD models from the weights of the Gaussian fits to stimulation sites. Data shown for the somatosensory ROI (S1, post-central gyrus) only. The white dot shows the peak value on the grid. A) Data separated by proximal-distal (PD location; column of preferred responses indicating digit, and rows indicating PD1 (tip) to PD4 (base). B) Data separated Brodmann area (BA); column of preferred responses indicating digit, and rows indicating BA3a to BA2.

**Supplementary Figure 5**: pRF size separated by Brodmann area across each digit. The grey line connects the median of each box and whisker chart. Asterisks indicate significant differences (p<0.05). Each point on the box-whisker charts is the mean from an ROI for a given subject. The pRF coverage maps are shown beneath for each model and are peak aligned. **A)** 2D Gaussian model with within-digit and between-digit directions shown**,** and **B)** 1D Gaussian WD and 1D Gaussian BD models.


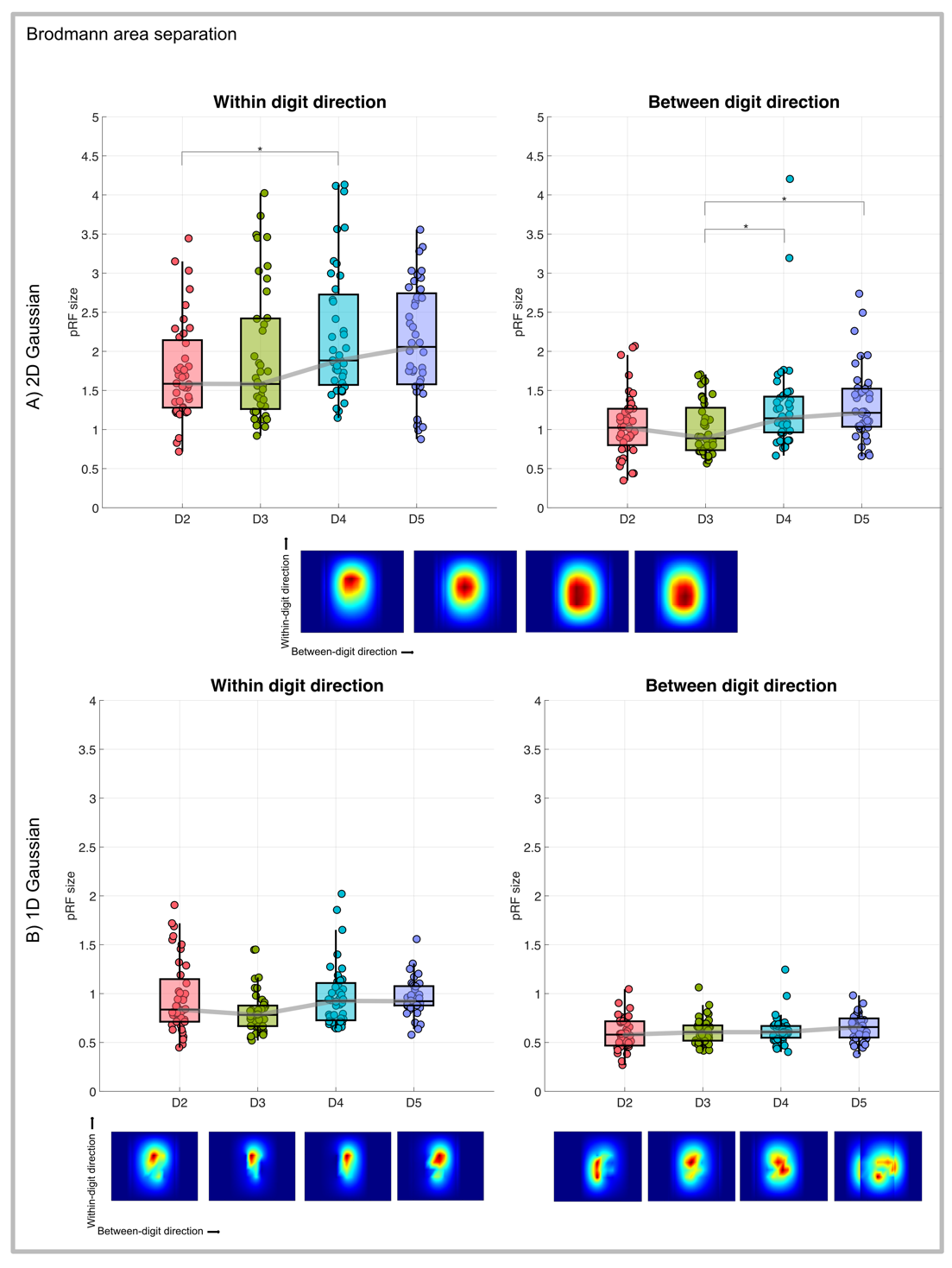


**
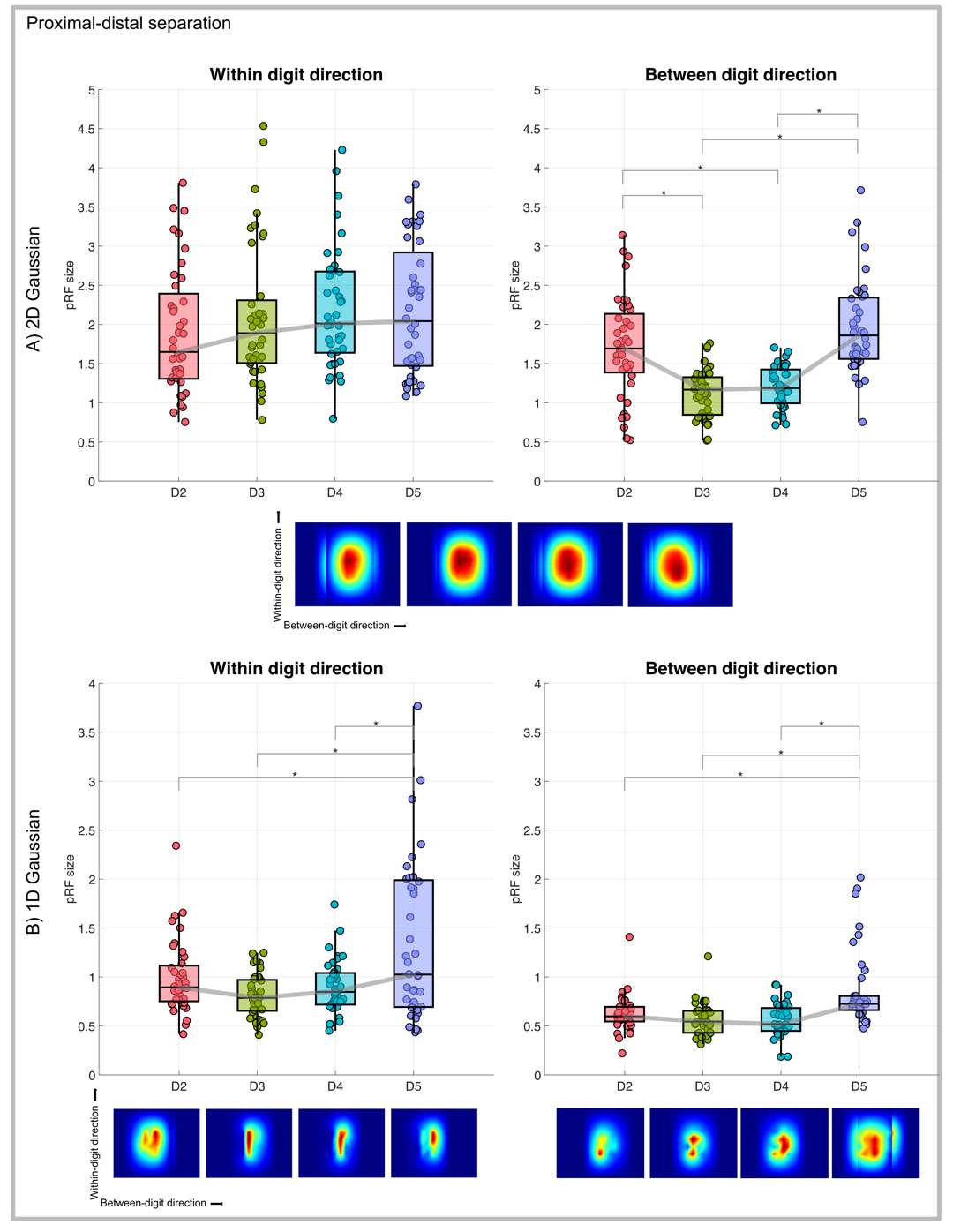
Supplementary Figure 6:** pRF size for each digit separated by proximal-distal location. The grey line connects the median of each box and whisker chart. Asterisks indicate significant differences (p<0.05). Each point on the box-whisker charts is the mean from an ROI for a given subject. The pRF coverage maps are shown beneath for each model and are peak aligned. **A)** 2D Gaussian model with within-digit and between-digit directions shown**,** and **B)** 1D Gaussian WD and 1D Gaussian BD models.

**Supplementary Table 1:** Number of parameters for each pRF model, all models include five haemodynamic response function (HRF) parameters.

| Models | Parameters | HRF parameters | Total number of parameters |
| --- | --- | --- | --- |
| 2D Gaussian | Mean of Gaussian (*x*)  Mean of Gaussian (*y*)  Standard deviation of Gaussian (*x*)  Standard deviation of Gaussian (*y*) | Timelag of HRF rise  τ_1_ (width of HRF)  Amplitude of undershoot  Timelag of undershoot  τ_2_ (width of undershoot) | 9 |
| 1D Gaussian BD  1D Gaussian WD | Mean of Gaussian x4  Standard deviation of Gaussian x4  Amplitude of Gaussian x3 *(One amplitude fixed for reference)* |  | 16 |
| Unconstrained | Free parameters x16 |  | 21 |

**Supplementary Table 2:** ANOVA on measures of pRF size for 2D and 1D models shown in Figure 7. Significant differences (p<0.05) that survive multiple comparisons correction shown.

**A)** Digits as a factor, with digits separated either by BA or PD location.

| **Model** | **Direction** | **Separation** | **F** | **Prob>F** | **Multiple comparisons survival (p<0.05)** | **Multiple comparisons p-value** |
| --- | --- | --- | --- | --- | --- | --- |
| 2D Gaussian | Within-Digit | PD(1,2,3,4) | 33.223 | 0.000 | PD1>PD2 | 0.000 |
|  |  |  |  |  | PD1>PD3 | 0.000 |
|  |  |  |  |  | PD1>PD4 | 0.000 |
|  |  |  |  |  | PD4>PD2 | 0.000 |
|  |  |  |  |  | PD4>PD3 | 0.021 |
|  | Between-Digit | BA(3a,3b,1,2) | 10.272 | 0.000 | BA2>BA3a | 0.000 |
|  |  |  |  |  | BA2>BA3b | 0.000 |
|  |  |  |  |  | BA2>BA1 | 0.001 |
|  |  |  |  |  |  |  |
| 1D Gaussian | Within-Digit | PD(1,2,3,4) | 3.507 | 0.017 | PD4>PD2 | 0.008 |
|  | Between-Digit | BA(3a,3b,1,2) | 2.905 | 0.037 | BA2>BA3b | 0.021 |

**B)** Proximal Distal (PD) locations or Brodmann Areas (BA) as factors

| **Model** | **Direction** | **Separation** | **F** | **Prob>F** | **Multiple comparisons survival (p<0.05)** | **Multiple comparisons p-value** |
| --- | --- | --- | --- | --- | --- | --- |
| 2D Gaussian | Within-Digit | BA(3a,3b,1,2) | 2.862 | 0.039 | D4>D2 | 0.037 |
|  | Between-Digit | PD(1,2,3,4) | 28.216 | 0.000 | D2>D3 | 0.000 |
|  |  |  |  |  | D2>D4 | 0.000 |
|  |  |  |  |  | D5>D3 | 0.000 |
|  |  |  |  |  | D5>D4 | 0.000 |
|  |  | BA(3a,3b,1,2) | 4.730 | 0.003 | D4>D3 | 0.028 |
|  |  |  |  |  | D5>D3 | 0.014 |
|  |  |  |  |  |  |  |
| 1D Gaussian | Within-Digit | PD(1,2,3,4) | 9.375 | 0.000 | D5>D2 | 0.003 |
|  |  |  |  |  | D5>D3 | 0.000 |
|  |  |  |  |  | D5>D4 | 0.000 |
|  | Between-Digit |  | 13.555 | 0.000 | D5>D2 | 0.000 |
|  |  |  |  |  | D5>D3 | 0.000 |
|  |  |  |  |  | D5>D4 | 0.000 |
